# Rare Skeletal Complications in the Setting of Primary Hyperparathyroidism

**DOI:** 10.1155/2015/139751

**Published:** 2015-11-17

**Authors:** Nikos Sabanis, Eleni Gavriilaki, Eleni Paschou, Asterios Kalaitzoglou, Dimitrios Papanikolaou, Pinelopi Ioannidou, Sotirios Vasileiou

**Affiliations:** ^1^Department of Nephrology, General Hospital of Pella, 58200 Edessa, Greece; ^2^Medical School, Aristotle University of Thessaloniki, 54124 Thessaloniki, Greece; ^3^Department of General Practice & Family Medicine, General Hospital of Pella, 58200 Edessa, Greece; ^4^Department of General Surgery, General Hospital of Pella, 58200 Edessa, Greece

## Abstract

Parathyroid carcinoma represents an extremely rare neoplasm with diverse clinical manifestations which vary from asymptomatic patients to severe complications of hypercalcemia or parathyrotoxicosis while skeletal involvement is rather common. Herein we aimed at presenting a unique case of a young patient with rare aggressive skeletal complications of parathyroid cancer that initially were misdiagnosed. Ossification of the cervical ligamentum flavum and skull tumor illustrates erosive bonny lesions of hyperparathyroidism that in association with previous medical history of recurrent nephrolithiasis and biochemical findings guide the diagnosis. We suggest that increased awareness and holistic approach are needed in order to recognize and further investigate signs and symptoms of hyperparathyroidism.

## 1. Introduction

Parathyroid carcinoma represents an extremely rare neoplastic entity, accounting for approximately 1% of primary hyperparathyroidism (HPT). Although hormonally functional tumors are observed in the majority of cases, clinical manifestations of parathyroid carcinoma can vary from none (asymptomatic patients) to severe complications of hypercalcemia or parathyrotoxicosis. Thus, its diagnosis remains a challenge for the clinicians and is primarily based on laboratory and imaging testing [1].

Herein we aimed at presenting a unique case of a young patient with severe complications of parathyroid cancer and briefly reviewing the relevant literature.

## 2. Case Report

A 45-year-old, Greek, Caucasian, male patient presented to the Emergency Department due to severe, colicky pain in the left pleura reflected to ipsilateral lower abdomen quadrant accompanied by nausea and vomiting. His personal medical history included recurrent episodes of nephrolithiasis, laminectomy in the cervical spine due to ossification of the cervical ligamentum flavum in C2-C3 and C4-C5 without signs of myelopathy two years ago, and surgical resection of a giant cell tumor of the skull one year ago. No familial history of multigland disease or evidence of hypercalcemia in his relatives was recorded.

Based on the clinical examination and renal ultrasonography the patient was diagnosed with another episode of nephrolithiasis without evidence of obstructive uropathy. Beyond that, however, laboratory testing revealed findings of primary hyperparathyroidism (serum calcium 16.0 mmol/L with normal values 8.0–10.4 mmol/L, phosphorus 1.46 mg/dL with normal values 2.5–5.9 mg/dL, parathyroid hormone 8560.0 pg/mL with normal values 8.0–76.0 pg/mL, and urine calcium levels 1260 mg/24 h). It is noteworthy that, on admittance, biochemical testing revealed also acute kidney injury (serum creatinine levels 1.76 mg/dL with normal range 0.5–0.9 mg/dL). Thus, the patient was hospitalized for further diagnostic procedures.

**Figure 1:**
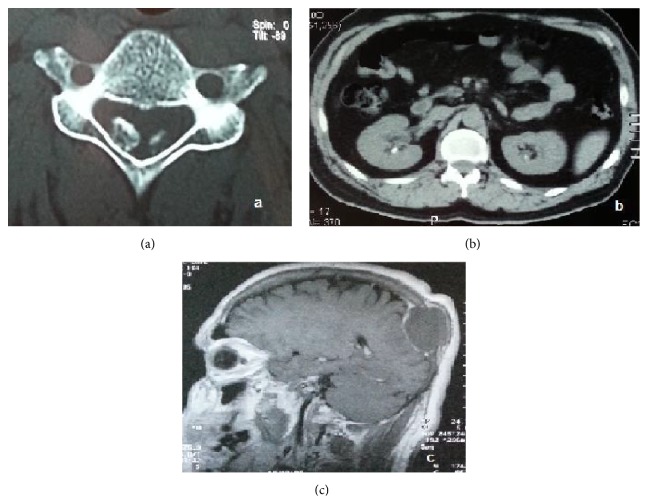
A 45-year-old man with parathyroid carcinoma: (a) ossification of the cervical ligamentum flavum in C2-C3 and C4-C5 without signs of myelopathy. (b) Nephrolithiasis in both kidneys and (c) left parietal bone tumor (5.5 × 3.2 × 4.4 cm) from Magnetic Resonance Imaging scan.

**Figure 2:**
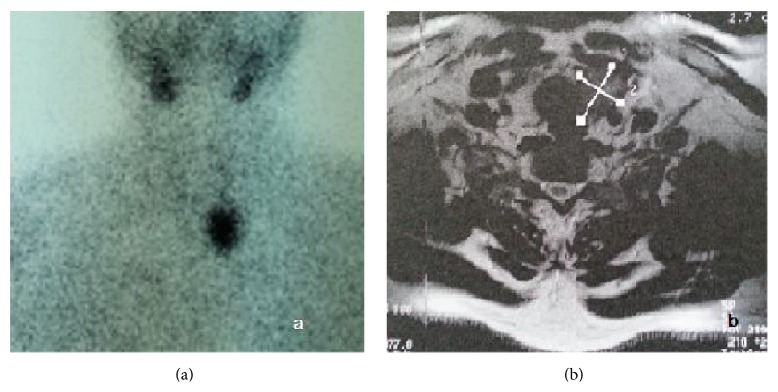
A 45-year-old man with parathyroid carcinoma: (a) Technetium-99m Sestamibi scan and (b) Magnetic Resonance Imaging scan showing the parathyroid tumor.

During his hospitalization the patient's history and medical records were carefully reviewed. As shown in Figure 1, the patient had been suffering from misdiagnosed complications of hyperparathyroidism for the last two years. Based on his history, neck ultrasound and Technetium-99m Sestamibi scan were performed revealing a parathyroid tumor, as shown in Figure 2. Thorax and abdomen Computed Tomography were performed revealing no further pathological findings as well as neck Magnetic Resonance Imaging. No genetic testing was performed, in spite of the young age of the patient, because of the absence of familial history of multigland disease as well as clinical and imaging findings compatible to an inherited form of hyperparathyroidism [2]. Due to the persistently high serum calcium and parathyroid hormone levels, the high alkaline phosphatase levels (440.0 IU/L with normal values 38.0–155.0 IU/L), and the late complications of hyperparathyroidism, surgical excision of the tumor was scheduled. Meanwhile, the patient was treated with intravenous administration of normal saline 0.9% and renal adapted dosage of zoledronic acid (3 mg, MDRD eGFR = 52 mL/min/1.73 m^2^). As a result, preoperative serum calcium and creatinine levels were improved. According to the histopathology the tumor was identified as parathyroid carcinoma and total surgical excision was achieved.

After surgery serum calcium and phosphorus levels were closely monitored in order to prevent potential hungry bone syndrome. The clinical course was uneventful and the patient remains on a regular follow-up program with no signs of recurrence or metastasis one year after the excision.

## 3. Discussion

In our case report we describe the coexistence of rare late complications of hyperparathyroidism, such as recurrent nephrolithiasis, ossification of the cervical ligamentum flavum, and skull brown tumor which had not been adequately investigated at their onset.

The clinical appearance of parathyroid carcinoma is diverse and not pathognomic. Skeletal involvement is rather common in parathyroid carcinoma (22–91%) [3]. It primarily includes diffuse osteopenia, osteoporosis, or pathological fractures as well as osteitis fibrosa cystic, subperiosteal bone resorption and absence of the lamina dura. Brown tumors had been extensively described as radiological features in 4.5–24% of patients with primary hyperparathyroidism [4, 5]. These erosive osseous lesions are observed in the setting of osteitis fibrosa cystic due to rapid osteoclastic activity. In the past decades, brown tumors were considered prominent manifestations of primary hyperparathyroidism [6, 7] or severe secondary hyperparathyroidism in hemodialysis patients [8]. Furthermore, ossification of the cervical ligamentum flavum has mainly been correlated to mechanical stress, growth factors, and trauma in exceptional limited case reports in Caucasian people [9].

Our patient had a medical history of atypical skeletal complications of hyperparathyroidism which had not been elucidated properly: a skull brown tumor, ossification of the cervical ligamentum flavum, and ligament calcification of the knee joint. Thus, he experienced two surgical operations. The histopathology of the skull brown tumor was initially misdiagnosed as a giant cell tumor of the bone and the previous medical history had been ignored. Of note, such misdiagnoses are also evident in the recent literature [10–12] but no previous report of ossification of the cervical ligamentum flavum in patient with hyperparathyroidism of any cause has been reported.

With regard to the renal manifestations of severe hyperparathyroidism we observed a history of recurrent episodes of nephrolithiasis in the context of excessive hypercalciuria. Silverberg et al. refer that calcium stone disease remains the most common clinical manifestation of primary hyperparathyroidism, ranging between 15 and 20% in most series. About 3% of patients with stone disease have primary hyperparathyroidism, and about 10% of patients with primary hyperparathyroidism present with recurrent calcium stone disease [13].

Beyond the high index of clinical suspicion, our patient presented also with extremely increased calcium, parathyroid hormone, and alkaline phosphatase levels. Therefore, the recommended imaging approach for the diagnosis of parathyroid cancer, which combines Technetium-99m Sestamibi scan and a neck ultrasound [1], proved to be important. Thorax, neck, and abdomen Computed Tomography as well as Magnetic Resonance Imaging scans were also advisable and were performed in order to exclude metastatic lesions due to the observed severe clinical manifestations. Complete tumor resection was achieved as confirmed by the histopathology. Normalization of calcium and parathyroid hormone levels was observed postoperatively without hungry bone syndrome appearance, persisting one year after initial surgery. Taking into consideration the high recurrence (more than 50%) and metastatic rate of the disease (approximately 25%) [1], long-term regular follow-up visits should be performed.

## 4. Conclusions

Parathyroid carcinoma is a rare neoplasm with diverse clinical manifestations. Since the patients are often referred to primary care physicians, general surgeons, orthopedic surgeons, or neurosurgeons for their initial symptoms, increased vigilance is needed in order to recognize and further investigate signs or symptoms mimicking those observed in hyperparathyroidism.
